# Primary care physiotherapists ability to make correct management decisions – is there room for improvement? A mixed method study

**DOI:** 10.1186/s12875-021-01546-1

**Published:** 2021-10-06

**Authors:** Cecilie Rud Budtz, Helle Rønn-Smidt, Janus Nikolaj Laust Thomsen, Rikke Pilegaard Hansen, David Høyrup Christiansen

**Affiliations:** 1 Department of Occupational Medicine, University Research Clinic, Gl. Landevej 61, 7400 Herning, Denmark; 2 VIA University College, Aarhus, Denmark; 3 Center for General Practice at Aalborg University, Aalborg, Denmark; 4 Central Denmark Region, Viborg, Denmark; 5 Department of Clinical Medicine, Health, Aarhus University, Aarhus, Denmark

**Keywords:** Management decision, Primary care physiotherapy, Musculoskeletal conditions

## Abstract

**Background:**

With increasing interest in direct access to physiotherapy, it is important to consider the physiotherapists (PTs) ability to make correct management decisions, because identification of differential diagnostic pathologies and timely referral for specialist care is vital for patient safety.

The aims of the study were to investigate PTs ability to make correct management decisions in patients presenting with musculoskeletal conditions and to identify explanatory factors associated with this ability. Furthermore, we wanted to explore the PTs views on the identified factors.

**Methods:**

The study was a mixed methods study with an explanatory sequential design consisting of a questionnaire survey and semi-structured interviews. The questionnaire comprised 12 clinical vignettes describing patient scenarios for musculoskeletal conditions, non-critical medical conditions and critical medical conditions. Based on this, the PTs indicated whether the patient should be managed by the PT or were in need of medical referral. Associations between correct decisions and explanatory variables was analyzed by mixed- effects logistic regression. Interviews were performed with nine PTs to explore their reactions to the results. A directed content analysis was performed.

**Results:**

A total of 195 PTs participated in the questionnaire survey and 9 PTs were interviewed. Overall, PTs were more likely to make correct management decisions in the musculoskeletal conditions category, whereas wrong decisions were more often chosen for underlying medical conditions categories. Positive associations between correct management decision in the critical medical category were found for experience: odds ratio (OR) 2.73 (1.33;5.57) and passed quality audit OR 2.90 (1.50;5.58). In the interviews, PTs expressed concerns about the differential diagnostic abilities. They all noted, that experience is immensely important in the clinical reasoning process because the ability to recognise diagnostic patterns evolves over time. Furthermore, the quality audit seems to address and systematize the clinical reasoning process and workflow within the clinics.

**Conclusion:**

The lack of ability to make correct management decision in critical medical categories and the uncertainties expressed by PT’s should raise concern, as direct access to physiotherapy is already well-established and the results indicate that patient safety could be at risk. The findings that experience and passed quality audit was associated with correct management decisions highlights the need for ongoing awareness and education into differential diagnostics.

**Supplementary Information:**

The online version contains supplementary material available at 10.1186/s12875-021-01546-1.

## Background

Musculoskeletal conditions are the single largest contributor to years lived with disability worldwide and as a natural consequence musculoskeletal conditions are one of the leading causes for care seeking in primary care [1, 2]. Primary care physiotherapy is often a central part of the treatment pathway for patients with musculoskeletal conditions, and is often based on referral from a General Practitioner (GP). It is anticipated, that primary care physiotherapy will play an even larger role in the treatment pathway in the coming years. This because direct access to physiotherapy has the potential to decrease the workload for GPs, decrease health costs as well as reduce delays in assessment and management [3].

With increasing interest in direct access to physiotherapy, it is important to consider the PTs abilities to make correct management decisions. The PTs ability to identify differential diagnostic pathologies and secure timely referral for specialist care is vital for patient safety, because most of the serious pathologies can be managed and treated efficiently if diagnosed early [4]. These abilities are a central part of the clinical reasoning process, which is a complex reflective process were diagnostic hypotheses are developed and tested to strengthen the decision-making process [5–9]. This also includes the ability to form differential diagnostic hypotheses and knowing precautions and contraindications for physiotherapy treatment, which also involves screening for serious pathologies that requires medical management [9]. Although clinical guidelines recommend screening for serious pathology as a natural part of the initial assessment of the patient, little is known about the PTs ability to screen and their confidence in doing it. The limited knowledge on physiotherapists ability to screen for serious diseases is one of the leading objections against direct access [3]. This objection is expressed by GPs as well as PTs, and the objection is not only based in limited knowledge on PTs abilities but also expressed concerns about the added complexity and responsibility as a first-in-line assessor [10, 11].

With that in mind, surprisingly few studies have investigated the PTs ability to make correct management decisions as well as their knowledge on screening for serious medical conditions. The conclusions in the published studies are however consistent; PTs have difficulties in detecting, asking for and documenting signs and symptoms of medical conditions [12–16], which consequently affects their management decisions [14, 17]. This stresses the need for further investigations into physiotherapists ability to screen for serious medical conditions. Also, previously conducted studies have mainly had a quantitative focus, where reasons and nuances as to why identified factors could be of importance when making correct management decisions have not been explored further. Combining both quantitative and qualitative methods, has the potential to inform the discussions into how management decisions could be enhanced among PTs and also enlighten the further considerations on direct access to physiotherapy.

Therefore, the aims of the study were to investigate PTs ability to make correct management decisions in patients presenting with musculoskeletal conditions and to identify explanatory factors associated with this ability. Furthermore, we wanted to explore the PTs views on the identified factors and why they are of importance.

## Methods

### Study design

The study was a mixed methods study with an explanatory sequential design [18]. The study consisted of two phases; a quantitative cross-sectional questionnaire survey and qualitative semi-structured interviews. The study was reported according to the STROBE checklist for cross sectional studies [19] and the Good reporting of A Mixed Methods Study (GRAMMS) guideline [20]. The qualitative phase was conducted to help explain and elaborate the results from the quantitative phase, the rationale being that the quantitative results provide a general understanding and the qualitative phase refines the results and explores the PTs views and experiences in more depth [18]. In this study, emphasis was given to the quantitative phase.

Integration occurred in two steps; the first step was to use the quantitative results to inform the qualitative interviews and the second to integrate the two sets of connected results and draw integrated conclusions.

Written and oral informed consent to participate in the study was obtained from all participants. The study was approved by the Danish Data Protection Agency (No. 1–16–02-41-19).According to Danish law, this study did not need ethics approval (Act on Research Ethics Review of Health Research Projects, October 2013) [21].

### Quantitative methods

#### Participants and setting

In Denmark, GPs act as free of charge gateways to the healthcare system with the overall responsibility for referral to primary and secondary healthcare. Hence, GPs can refer patients to primary care PT with approximately 40% reimbursement. However, direct access to primary care PT is also a possibility (through private healthcare insurance schemes or off-the-street treatment without reimbursement), which does not prerequisite any additional education or certification for the PTs.

A total of 60 primary care PT clinics from Central Denmark Region (one of five Regions in Denmark) were contacted through email and follow-up telephone calls and invited to participate in a questionnaire survey. The clinics were randomly selected with a 1:1 rate on clinic size, meaning 30 small (six PTs or less) and 30 large (more than 6 PTs) were invited. A total of 27 clinics agreed to participate (45%). To ensure high participation rates and avoid selection problems, the decision was made to physically visit the clinics who agreed to participate in the survey. The clinics were all visited from August through October 2020 and during the visit all PTs at the clinic were encouraged to participate in the survey. It was emphasized verbally during the visit, that participation was voluntary and that the PT could decline to participate. The questionnaire was completed electronically by mobile phone. The PTs were not allowed to talk with each other during completion of the survey. Questionnaire data was collected using the RedCap system [22].

##### Development and pilot test of questionnaire

The questionnaire comprised two separate sections. The first section included background information on each PT; gender, age, years of clinical experience in private practice, training and education the past 5 years, which type of patients the PT treated and how large a proportion of the patients they treated without referral from the GP. Also, the PTs were asked if they would be willing to participate in a follow-up interview.

**Case vignettes**

The second section of the questionnaire included 12 short clinical vignettes. The use of clinical vignettes is recognized as a valid method for measuring variations in clinical decision making abilities [23]. The vignettes described a hypothetical patients age, gender and the clinical presentations for which the patient sought the PT for assessment and treatment. The vignettes were based on previously developed and validated vignettes [17]. The vignettes described clinical presentations that were either medical conditions, which should not be treated by a PT alone, or musculoskeletal conditions, which would be appropriate for the PT to manage without consulting the patients GP. The medical conditions could be either non-critical or critical based on the urgency for further medical attention. Based on the description, the PTs were asked to make a management decision. There were three possible choices of management; A) to provide physiotherapy intervention, B) to provide physiotherapy intervention while encouraging the patient to contact their GP for further assessment and C) no physiotherapy intervention and a direct referral to the GP.

The original vignettes were developed to describe physiotherapists’ ability to make management decisions on physiotherapy intervention or medical referral in a direct-access setting. In this study, the PTs were not to assess the vignettes in a solely direct-access setting, as Danish primary care physiotherapy embrace both direct-access (without reimbursement) and access through referral (with approximately 40% reimbursement). To ensure the vignettes were appropriate in Danish context, the original vignettes were translated into Danish and additionally six vignettes were developed to ensure a broad range of musculoskeletal conditions were represented. The vignettes were reviewed and revised by an consensus group consisting of 2 GPs and 4 experienced practicing PTs (all had been practicing for more than 10 years) using the Nominal Group Technique [24]. The group was initially presented to 7 musculoskeletal (MS), 6 non-critical medical (NCM) and 5 critical medical (CM) vignettes. First, the group was asked to read the vignettes individually, note any comments on content or unclearness and finally rate each vignette from 1 to 5 in relation to relevance and credibility. Afterwards, the rating was reviewed by the whole group and consensus on which vignettes to include was reached. A total of 5 MS, 4 NCM and 3 CM vignettes were included in the final questionnaire (see Additional file 1).

**Pilot testing**

The final questionnaire was pilot tested in a sample (*n* = 7) of PTs. The PTs were interviewed using the cognitive techniques *think-a-loud interviewing* and *verbal probing* to ensure the questionnaires comprehensibility and comprehensiveness [25]. Only minor revisions were needed after the pilot test.

##### Correct management decision

Each of the three response categories was dichotomized into correct management decision (yes/no). For *musculoskeletal conditions* correct management was; A) to provide physiotherapy intervention or B) to provide physiotherapy intervention while encouraging the patient to contact their GP for further assessment, whereas incorrect management decision was C) no physiotherapy intervention and a direct referral to the GP. For *non-critical medical conditions* correct management was; B) physiotherapy intervention while encouraging the patient to contact their GP for further assessment or C) no physiotherapy intervention and a direct referral to the GP. *For critical medical conditions* correct management was; C) no physiotherapy intervention and direct referral to the GP.

The main outcome was the PTs ability to correctly identify the correct management decision regarding the group of *musculoskeletal*, *non-critical medical* and *critical medical* conditions. Making a correct management decision is a complex reasoning process, and often the correct answer to the vignettes was debatable, especially in the medical conditions categories. To account for this, different outcomes were defined for the categories of conditions. Hence, correct management decision for the musculoskeletal conditions was defined as five correct answers to the five MS vignettes. We defined correct management decision for the non-critical medical conditions as three correct answers to the four NCM vignettes and finally for critical medical conditions as two correct answers to the three CM vignettes.

##### Explanatory variables

The explanatory variables were a priori chosen primarily based on previously conducted studies [14, 17] and all variables were self-reported:

1) Experience; years of experience as a primary care physiotherapist, 2) specialization; defined as completed and certified MDT (McKenzie Method), MT (Musculoskeletal Specialization) or CMP (Certified Mulligan Practitioner) physiotherapists, 3) treating patients without referral; PTs indicated whether or not they already treat patients without referral from the GP and 4) passed quality audit; A nationwide quality audit commenced in 2019, which includes all primary care physiotherapy clinics with the purpose of evaluate and develop the quality of primary care physiotherapy [26]. Among pre-specified indicators of quality is an evaluation of the written patient record including evaluation of red flags (signs or symptoms of serious pathology) [27].

##### Sample size

Based on previously conducted studies [12, 17], it was anticipated that there would be a 20%-point difference between the highly experienced versus the less experienced PTs abilities to make correct management decisions in the critical medical vignettes. To detect a 20%-point difference (power 0.80, alpha 0.05) a total of 194 PTs were needed. The 27 clinics who agreed to participate employed 239 PTs and with an expected participation rate of 85% the needed number would be reached.

#### Qualitative methods

The qualitative phase consisted of semi-structured interviews with primary care PTs. We made a purposeful sampling of PTs among those who had consented to participate in an interview. We included PTs who made correct as well as incorrect management decisions. Also, we wanted a broad range of PTs representing the different explanatory variables, e.g. PTs with few as well as many years of experience. This enabled different views and experiences to inform the qualitative phase.

An interview guide was developed by CRB and HRS based on the quantitative results. The guide focused on the PTs reactions to the results and their experience in relation to management decisions (se interview guide in Additional file 2). The PTs were not confronted with their answers to the questionnaire during the interview, they were merely presented with the overall conclusions. The guide covered both open-end and follow-up probe questions. After the first two interviews were performed by CRB, transcripts were made and read through by CRB and HRS to ensure the questions were covering the aspects of the quantitative results as attended. Consequently, minor revisions were made to the interview guide. We invited 24 PTs to participate in the interviews, and 9 agreed to participate (PTs mostly declined due to time pressure). The interviews (lasting 30–45 min each) were performed online via ZOOM and recorded in January 2021 by CRB.

#### Data analysis

##### Quantitative data

Descriptive statistics (percentages, means) were used to characterize the participants and practice settings. Also, percentages of correct management decisions were calculated for each vignette as well as the three categories of conditions.

We then analyzed the association between correct management and explanatory variables for each of the three conditions using mixed effects logistic regression models, to ensure clustered data was handled correctly [28]. The dependent variable was correct management decision (yes/no) for the specific group of conditions (e.g. 5 out of 5 correct answers in the musculoskeletal conditions). The explanatory variables were clinical experience in private practice (divided into 0–5 years or 5+ years), specialization (yes/no), treating patients without referral (direct access) (yes/no) and if the clinic had passed a quality audit (yes/no). The analyses were presented as Odds Ratio (OR) with 95% Confidence Interval (95%CI) and adjusted for all other explanatory variables. Considerations on the number of explanatory variables to include in the analyses were based on the principle of at least 10 cases per variable.

All statistical analyses were performed using STATA version 16.0 (StataCorp LP, College Station, TX, USA).

##### Qualitative data

The interviews were transcribed verbatim. A directed content analysis was performed with a deductive approach, as described by Hsiu-Fang and Shannon [29]. The quantitative results were used as categories for coding the transcripts, meaning initial coding involved marking the transcripts based on the following five categories; 1) Ability to make correct management decision, 2) experience, 3) specialization, 4) direct access and 5) quality audit. Relevant quotes were presented to illustrate the analyses results.

All analyses were made using QSR International (1999) NVivo Qualitative Data Analysis Software version 12.

## Results

A total of 27 clinics participated, divided into 11 small clinics and 16 large clinics (> 7 PTs employed). Nine of the participating clinics had passed the quality audit. 195 PTs answered the questionnaire, equivalent to 82% of the PTs from the participating 27 clinics. The PT characteristics are presented in Table 1.

**Table 1:** Characteristics of participating physiotherapists

	n	%
Number of physiotherapists	195	
Gender, female	106	54
Age^a^	41	12
Years practised at clinic (experience)		
0–5 years	71	36
6–15 years	46	24
15 + years	78	40
Training and education the past five years^b^		
0	11	6
1–5	92	47
6–10	63	32
11+	29	15
Specialization	57	29
Direct access patients	152	78

Abbreviations: *n* number, *SD* standard deviation

^a^ Presented as: Mean and Standard Deviation (SD)

^b^ Divided into courses with varying subjects and lengths

### Management decisions

#### Quantitative results

In total, 82 PTs (42%) made a correct management decision regarding the musculoskeletal conditions (5 correct / 5 MS vignettes), 73 PTs (37%) regarding the non-critical medical conditions (3 correct / 4 NCM vignettes) and 67 PTs (34%) regarding the critical medical conditions (2 correct / 3 CM vignettes). Experienced PTs (> 5 years experience) more often made correct management decisions compared to less experienced; with a 11%-point difference in the musculoskeletal category (46% vs. 35%), a 4%-point difference in the non-critical medical category (39% vs. 35%) and finally a 23%-point difference in the critical medical category (43% vs. 20%). Also, the 5% answering 100% correct in the critical medical category (3 correct / 3 CM vignettes) were all experienced PTs. Looking at the PTs answers to the included 12 vignettes in Table 2, there was a clear tendency towards very similar and correct management decisions in the musculoskeletal category where over 90% of the PT made a correct management decision in four out of five vignettes. In contrast, a more miscellaneous and often wrong management decision was made in the medical conditions categories.

**Table 2:** Percentage of correct management decision

	PT	PT but encourage GP contact	No PT and GP referral	Correct MD	100% correct MD^**b**^
**Musculoskeletal conditions**				**42%**^**a**^	**42%**
Man with leg pain	74%	24%	2%	98%	
Woman with neck pain	88%	11%	1%	99%	
Woman with pain around sternum	6%	39%	55%	45%	
Girl with knee pain	51%	40%	9%	91%	
Man with knee pain	77%	22%	1%	99%	
**Non-critical medical conditions**				**37%**^**a**^	**5%**
Man with bilateral leg cramps	21%	67%	12%	79%	
Woman with foot pain	80%	15%	5%	20%	
Woman with bilateral shoulder pain	59%	34%	7%	41%	
Woman with intense subcostal pain	14%	36%	49%	86%	
**Critical medical conditions**				**34%**^**a**^	**5%**
Man with swollen and red knee	14%	37%	49%	49%	
Woman with intense low back pain	57%	27%	15%	15%	
Man with thoracic back pain	8%	36%	56%	56%	

Abbreviations: *CM* critical medical, *GP* General Practitioner, *PT* Physiotherapy, *MD* Management Decision, *MS* Musculoskeletal, *NCM* Non-critical medical

^a^ Defined as; musculoskeletal conditions (5 correct / 5 MS vignettes), non-critical medical conditions (3 correct / 4 NCM vignettes), critical medical conditions (2 correct / 3 CM vignettes)

^b^ Defined as; musculoskeletal conditions (5 correct / 5 MS vignettes), non-critical medical conditions (4 correct / 4 NCM vignettes), critical medical conditions (3 correct / 3 CM vignettes)

#### Qualitative results

Some of the PTs who participated in the interview were not surprised, that the answers diverged more in the medical conditions categories. As two of them expressed:

*“That probably agrees very well with our knowledge as physiotherapists. This (the musculoskeletal area) is the area we’re specialists in. And then there is perhaps more of a knowledge gap in the differential diagnostics.” [Male, 5 years of experience]*

*“Perhaps it is an indication of uncertainty among physiotherapists that we answer differently in the medical area. Whereas we know what we are talking about or agree in the musculoskeletal area.” [Female, 3 years of experience]*

Others were more surprised and also expressed concerns to the fact that management decisions differed in the medical conditions categories:

*“It actually does (surprise me)… Basically we ought to know more on these medical conditions so we could answer more similar.”* [Female, 11 years of experience]

### Explanatory variables

#### Quantitative results

Table 3 displays associations between correct management decision and explanatory variables for the three categories of conditions. Especially in the critical medical conditions category, it seems that the strongest associations were found between correct management decision and personal experience and passed quality audit.

**Table 3:** Associations between correct management decision and explanatory variables

	Musculoskeletal conditions		Non-critical medical conditions		Critical medical conditions	
	OR^a^	95% CI	OR^a^	95% CI	OR^a^	95% CI
Experience over 5 years	1.49	(0.80;2.76)	1.07	(0.57;2.01)	2.73	(1.33;5.57)
Specialization	1.28	(0.66;2.42)	1.32	(0.68;2.57)	1.90	(0.94;3.82)
Direct-access patients	1.04	(0.52;2.07)	1.24	(0.60;2.54)	1.15	(0.54;2.46)
Passed quality audit	1.09	(0.60;1.98)	1.89	(1.03;3.48)	2.90	(1.50;5.58)

^a^Adjusted for all other variables shown. Abbreviations: *CI* confidence interval, *OR* odds ratio

#### Qualitative results

Going into depth with variables associated with correct management decision, all the interviewed PTs found experience to be the most important factor when addressing correct management decisions. They pointed out, that recognizing diagnostic patterns, getting a gut-feeling that something is wrong and having experienced courses of treatments with patients suffering from medial conditions masquerading as musculoskeletal conditions are main reasons as to why experience forms the clinical reasoning process, especially around the medical conditions.

*“ We had this one (vignette) with pain around the thoracic spine and many would maybe think that if it is the thoracic area there’s probably nothing there. Now, I’ve treated a patient where there actually weren’t any red flags but after a long time we sent the patient back because nothing happens and it turns out to be spinal cancer… So you have that experience with you the next time… No matter how and how much you are educated and how much supervision you have, there are some things, you just can’t. It’s not until you are in it and you treat those patients you learn the clinical reasoning in these conditions.”* [Female, 4 years of experience]

With regards to specialization, the PTs overall thoughts were, that the different approaches to the patients in the different specializations had great impact on the answers to the vignettes and also to the clinical reasoning process in general. The PTs feel, that the different specializations offers a systematic approach to the examination, also in regards to possible differential diagnostic.

Already treating patients without referral showed no association with correct management decision, which also reflects the PTs experience in handling direct access patients. Their perception is, that the referral perhaps guides a little, but often the quality and information on the referral is very sparse, meaning they have to perform their examination as if they had no information. Some however also indicate, that their conversation with and examination of patients are more focussed when no referral exists.

*“I think that perhaps 50% of our patients are insurance cases. Most often they don’t have a referral. Maybe I’m a little more thorough asking questions and thinking possible differential diagnostic.”* [Male, 5 years of experience]

The detected association between passed quality audit and correct management decision initially surprised the PTs.

*“That’s a surprise to me. I think it’s a positive that you get more attention on some things. But I’m actually surprised it means so much.”* [Male, 1 year of experience]

Reflecting more on this, they could all se, that the quality audit has had a huge impact on their individual clinical reasoning process, as to structure, reflection and documentation of their findings. But also, the audit forced the clinic to address the PTs workflow in relation to screening for red flags and furthermore prompted cooperation between the PTs in discussing and defining how to reflect and react to red flag findings.

*“We’ve been through this process and I can see afterwards in our clinic we’ve all made more of an effort with the patient record. I think, we have become much more structured and we have included an item on clinical reasoning and hypothesis… I think we have become much more sharp.”* [Female, 14 years of experience]

## Discussion

This was a mixed method study examining primary care PTs decision making abilities and explanatory factors associated with this ability. Overall, PTs were more likely to make correct management decisions in the musculoskeletal conditions category, whereas they were more often wrong in the medical conditions categories. Especially among the critical medical conditions, associations between correct management decisions and experience and passed quality audit was detected. The quantitative results were further elaborated by qualitative results, where the interviewed PTs also thought experience was very important when making management decisions, because the clinical reasoning process evolves and develops over time. Furthermore, the quality audit had been an opportunity to address and systematize the clinical reasoning process and individual workflow as well as introducing more cooperation and discussions within the clinics.

### Strength and limitations

The cross-sectional survey has some limitations. The survey was researcher developed and the scenarios provided were very short. Although they were validated through a consensus group, making a correct management decision is a complex process, and the PTs would most probably collect additional data from the anamnesis, observations of the patient and a physical examination to inform their clinical reasoning in real life. Not having this possibility have of course affected the management decisions. Also, the PTs may have indicated different management decision to the vignettes than they would have made in clinical settings due to social desirability, that is answering the vignettes as they should have done instead of what they actually would have done in the clinical situation. This would introduce social desirability bias, which would overestimate the PTs abilities to make correct management decisions. We believe the selection bias in this study is limited, because high participation rates on the included clinics were obtained. Furthermore, non-participation would be random and not associated with neither explanatory variables nor the outcome. Likewise, any information problems with misclassifications of explanatory variables or management decisions would also be random and non-differentiated and could not explain the detected associations.

Due to the limited explanatory variables and sample size, it is likely that unmeasured variables such as personal preferences or cautions, composition of the PTs in the clinic, social capital or management of the clinics would have great impact on the work environment and workflow, thereby possibly affect the management decisions. This would be very interesting to explore in future studies.

The interviews were performed by a less experienced interviewer (CRB), but the data analyses were performed in collaboration with a highly experienced interviewer and qualitative researcher (HRS) thereby enhancing the credibility of the findings. As this was a study with a highly deductive approach, were the quantitative results framed the qualitative interviews, a natural dependability between the different research steps was present. During the interview- and analyses process, memos were used to record reflections and preconceptions to try to avoid a confirmatory approach to the interviews and analysis. The framing and nature of the directed content analysis however complicates neutral interview questions and some of the PTs may have answered questions in a certain way or agreed to please the interviewer. However, an effort was made to avoid this by stressing and recognizing how difficult and complex these differential diagnostic clinical reasoning processes are, thereby appreciating the PTs honest answers and justifying their expressed uncertainties in the area.

In this study, a mixed methods design was chosen to broaden the understanding of how and why the chosen explanatory variables was of importance when making correct management decisions. This enabled a more nuanced view on the management decisions as well as more informed interpretation of the quantitative results. In future studies investigating decision-making abilities, it would be highly valuable to further explore the mixed methods design options.

### Interpretation

In our study, we defined correct management decision in the medical conditions categories as 3 correct out of 4 NCM vignettes and 2 correct out of 3 CM vignettes to account for the complex reasoning process and debatable correct answers. This meant that 37 and 34% of the PTs made a correct management decision in the two medical conditions categories. If we had chosen 100% correct management decision, only 5% of the participating PTs would have achieved that. In major contrast, a directly comparable study by Jette and colleagues found that around 50% of the PTs answered 100% correctly in the medical conditions categories [17]. We however believe the 50% to be overestimated due to selection bias. Nevertheless, our results indicate clear discrepancies in management decisions in the medical conditions categories, and especially in the critical medical conditions category it seems alarming that many PTs indicated physiotherapy intervention without any need for GP assessment. Furthermore, Jette and colleagues did not find an association between correct management decision and experience (over 10 years) [17]. In our study, we found an association between correct management decision and experience. We however chose to make a cut-off of 5 years, which we believe is a better cut off to achieve a contrast between novice and experienced PTs. Our results also showed that only 20% of the less experienced PTs made correct management decisions in the critical medical category. These findings could call for more systematic training of newly qualified physiotherapist, as well as more attention to differential diagnostics at pre-graduate level. This is supported by another similar study examining decision-making abilities of final year undergraduate physiotherapy students. The study concludes, the students were not sufficiently equipped with knowledge and skills to make precise management decisions, and this was especially the case among critical medical conditions [30]. Jette and colleagues found an association between correct management decision and orthopaedic specialization [17]. Similar results were found by Ladeira that concluded, that PTs with for example orthopaedic specialization performed significantly better in making correct management decisions [14]. In our study, we included three different specializations; MT, MDT or CPM, but could not identify a significant association. This could indicate, that the specializations currently offered in primary care physiotherapy is not enough to secure management decision abilities. Nevertheless, specialization was thought to be of great importance to management decisions among the PTs because these specializations offer a further introduction into differential diagnostic pathologies among musculoskeletal patients and to some extend a systematised approach to screening for serious pathologies. In this study, we have not considered the timing of the specialization, meaning some of the PTs have perhaps completed the specialization several years ago, and therefore their possible advantage in differential diagnostics may be time limited. This could suggest a need for ongoing awareness and education into differential diagnostics that goes beyond specialization. Adding to this argument is the found association between management decision and passed quality audit, because the quality audit is a current event, meaning the clinics who have already passed the audit have very recently addressed clinical reasoning and screening for serious pathologies in the clinics. This emphasizes the need for ongoing awareness and education into these differential pathologies, because revisiting the clinical reasoning in these situations have great learning potential. Also, the fact that the audit forced the clinic to cooperate and reflect with each other has a huge potential to further develop the PTs abilities in this area. Also, it is worth noticing that the quality audit is a modifiable factor as supposed to experience, which we cannot modify.

Taking the results from this study into consideration combined with the fact that direct access is already a well-existing possibility in Denmark should however raise some concern; Almost 80% of the participating PTs treated direct access patients regardless of their experience, specialization status or whether or not the clinic had passed the quality audit. This stresses the need for further investigation into this area, because at present newly educated PTs can examine and treat patients in direct access and the results from this study indicates, that patient safety may be threatened in such situations. To address this, an increased focus on or demand for supervision and/or collaboration with colleagues and GPs could perhaps help newly educated PTs in these direct access situations. Nevertheless, PTs as first-in-line examiners of musculoskeletal conditions could also be seen as an opportunity to ensure early identification of serious medical conditions, because PTs often have the patients in longer courses of treatment which gives them a unique possibility to discover the often fluctuating and diverse symptoms of serious pathologies. This however prerequisites further awareness into the area of differential diagnostics and the PTs abilities and knowledge on screening for serious pathologies.

### Generalisability

The invited clinics were all located in Central Denmark Region. The Region is the second largest out of five regions in Denmark and we believe this region to be representative to the rest of Denmark with respect to geography, general population and the dispersion of physiotherapy clinics. We therefore believe our results to be generalizable to PTs in similar healthcare systems and with similar educational level. We however also recognise, that both healthcare systems and the education of PTs differ greatly from country to country.

## Conclusion

The PTs decision-making abilities leaves room for improvement; PTs were more likely to make correct management decisions in the musculoskeletal conditions category, whereas they were more often wrong in the medical conditions categories. The lack of ability to make correct management decision in critical medical categories and the uncertainties expressed by PTs should raise concern, as direct access to physiotherapy is already a well-established possibility and the results indicate that patient safety could be at risk. Experience and passed quality audit was strongly associated with correct management decisions among PTs. These results calls for more systematic training of newly qualified physiotherapist as well as ongoing awareness and education into differential diagnostics that goes beyond postgraduate specialization.

## Supplementary Information

**Additional file 1.**

**Additional file 2.**

## Data Availability

The study dataset from which we have reported findings in this paper cannot be accessed by other researchers according to Danish regulations. Access to similar data can be applied through Statistics Denmark.

## Abbreviations

CI: Confidence Interval
CM: Critical medical
CMP: Certified Mulligan Practitioner
GP: General Practitioner
MDT: McKenzie Method
MS: Musculoskeletal
MT: Musculoskeletal Specialization
NCM: Non-critical medical
OR: Odds Ratio
PT: Physiotherapist
SD: Standard Deviation
