# Supplementary material for: Primary care physiotherapists ability to make correct management decisions – is there room for improvement? A mixed method study
Source: BMC Fam Pract. 2021 Oct 6;22:196. doi: 10.1186/s12875-021-01546-1 (PMC8496017; doi:10.1186/s12875-021-01546-1)
Supplement: Supplementary file 1 — Additional file 1. [file 12875_2021_1546_MOESM1_ESM.docx]

**Additional file 1**

|  | | | |
| --- | --- | --- | --- |
| **MUSCULOSKELETAL CONDITIONS** | | | |
|  | **CASE** | **VIGNETTE** | **CLINICAL REASONING** |
|  | Man with leg pain | A 45-year-old man complains of consistent pain in his right leg, especially around the thigh. He is a builder and have had heavy and physically demanding work for the past 25 years. In the past couple of years, he has had episodes of low back pain. The pain had a sudden onset, but he does not recall a precipitating incident or injury. There is decreases ROM in the lower back, especially in flexion to the right side which also aggravates the pain. | Symptoms could indicate referred pain from a slipped disc (L4) in the related dermatome. |
|  | Woman with neck pain | A 39-year-old woman complains of 6-9 months of intermittent but increasing pain and tenderness around the neck area. There is local muscular tenderness and pain with movement. ROM of the neck is slightly decreased. There is no dizziness or radiation of pain. | Neck pain without red flags |
|  | Woman with pain around sternum | A 53-year-old woman with a fairly sedentary lifestyle complains of a sudden onset of deep and intense pain around the sternum. The pain increases with movement of the left arm. She cannot identify a precipitating incident or injury. The pain is not radiating, and there is extreme tenderness to palpation lateral to the sternum. Coughing and sneezing increase the pain. | The pain is aggravated by movement of the arm and tender to palpation. These symptoms are consistent with musculoskeletal origins of pain and no danger signals are reported. Could indicate costochondritis. |
|  | Girl with knee pain | A 17-year-old girl complains of knee pain following a football game where she stepped into a hole. She was unable to complete the remainder of the game. The medial aspect of the knee is tender to palpation and slightly swollen. The pain increases at the ends of ROM and with valgus stress. No complaints of knee locking. | Symptoms indicates a medial collateral ligament injury. |
|  | Man with knee pain | A 65-year-old man, former football player and current tennis player, complains of pain in his left knee, worsening over the past 6 months. The pain is affecting his leisure time activities as it increases with activity and movement. He reports a feeling of grinding in his knee. No swelling is noted, and the ROM is normal. | No red flags. Symptoms could indicate osteoarthritis. |
| **NON-CRITICAL MEDICAL CONDITIONS** | | | |
|  | Man with bilateral leg cramps | A 65-year-old man with a history of COPD and significant cigarette smoking complains of bilateral leg cramping associated with stair climbing beginning about 6 months ago. Over the past 2 months, the cramping has become associated with walking as well. When he sops walking and stands still, the cramping decreases and then disappears. No reflex or sensory changes are detected, and the pain is not affected by trunk flexion er extension. | The patient has multiple risk factors for cardiovascular disease; age, sex and smoking. Pain described with "cramping" that relieves with rest is classically described as claudication. |
|  | Woman with foot pain | A 35-year-old woman who is healthy and an occasional jogger complains of pain in the anterolateral aspect of the forefoot starting about a week ago. The pain started when she was running and is exacerbated by any weight bearing. There is moderate tenderness to palpation; no swelling or redness is noted. Foot and ankle ROM are normal. | Pain with palpation and weight bearing could indicate fracture of the fifth metatarsal. |
|  | Woman with bilateral shoulder pain | A 63-year-old woman complains of bilateral shoulder pain and stiffness. The pain has been increasing over the past months, and she feels it is worsening. She cannot identify a precipitating incident or injury. The pain typically decreases during the day. There is bilateral muscle weakness and limited ROM. | Increasing pain and bilateral stiffness is not consistent with musculoskeletal pain. Symptoms could indicate polymyalgia. |
|  | Woman with intense subcostal pain | A 55-year-old woman complains of constant, intense aching back pain subcostally on the right side over the past 2-3 days. The pain radiates along the iliac crest on the right side. She cannot identify a precipitating incident or injury. The pain is not affected by positional changes or the use of a heating pad. | The pain is not affected by positional changes and based on the pain location the symptoms could indicate visceral origin. |
| **CRITICAL MEDICAL CONDITIONS** | | | |
|  | Man with swollen and red knee | A 60-year-old man who apparently is healthy, but inactive, complains of sudden onset of pain in the right knee with no known precipitating incident. The joint is very tender, warm, and red. ROM is painful and decreased. No other previous or current joint complaints are reported. | Symptoms suggests a septic knee, arthritis or a joint affected by pseudogout. |
|  | Woman with intense low back pain | A 75-year-old woman complains of intense low back pain. The pain started while she was moving heavy boxes around. However, she cannot identify a precipitating incident or injury. The pain decreases with rest and is not radiating. There is extreme localized tenderness to palpation. | The patients age, intense pain and local extreme tenderness suggest a vertebra fracture. |
|  | Man with thoracic back pain | A 45-year-old man complains of mild-to-moderate thoracic back pain that is preventing his sleeping at night. The pain is intermittent but has increased over the past 2 weeks and is not relieved by positional changes. It seems to be worse at night than during the day. The patients complain of fatigue that he attributes to not sleeping well. | Red flags; the pain is not affected by positional changes, is slowly progressing, worse at night and preventing his sleeping and fatigue. These red flags suggest neoplasm. |
| Abbreviations: COPD = Chronic Obstructive Pulmonary Disease, ROM = Range of Motion | | | |
