# Supplementary material for: Primary care physiotherapists ability to make correct management decisions – is there room for improvement? A mixed method study
Source: BMC Fam Pract. 2021 Oct 6;22:196. doi: 10.1186/s12875-021-01546-1 (PMC8496017; doi:10.1186/s12875-021-01546-1)
Supplement: Supplementary file 2 — Additional file 2. [file 12875_2021_1546_MOESM2_ESM.docx]

**Additional file 2**

| **Semi-structured interview guide** | |
| --- | --- |
| **Main questions** | **Probe questions** |
| What are your initials thoughts to the conclusion that PTs give similar answers to musculoskeletal vignettes but different answers to the medical conditions? | What do you think is the reason for these different answers (experience, knowledge or something else)? |
| It looks like those who answer correctly are those with most experience (and the clinics that has passed the quality audit) – what are your thoughts on that? | Why is experience important?  What has the quality audit meant for your clinical practise and reasoning? |
| How was the experience of answering the questionnaire? Did it make you reflect on your own practise or did you discuss it in the clinic? | How do you handle screening for serious pathology among your patients? What do you do?  Does the existence of a medical referral change your approach to your patient? Why/ why not? |
| Have you ever tried having a patient in a course of treatment, which eventually turned out to have a serious pathology? Could you try and tell about the experience? | What made you react?  Did that change your approach to screening for serious pathologies afterwards? |
